# The relationship of the ratio of platelet distribution width to serum albumin with kidney disease progression in patients with hypertension

**DOI:** 10.1038/s41598-025-05575-z

**Published:** 2025-07-01

**Authors:** Kenichi Tanaka, Hiroshi Kimura, Hirotaka Saito, Michio Shimabukuro, Koichi Asahi, Tsuyoshi Watanabe, Junichiro James Kazama

**Affiliations:** 1 Department of Nephrology and Hypertension, Fukushima Medical University, 1 Hikariga-oka, Fukushima City, 960-1295 Fukushima Japan; 2 Division of Advanced Community Based Care for Lifestyle Related Diseases, Fukushima Medical University, Fukushima, Japan; 3 Department of Diabetes, Endocrinology, and Metabolism, Fukushima Medical University, Fukushima, Japan; 4 Division of Nephrology and Hypertension, Iwate Medical University, Yahaba, Japan

**Keywords:** Platelet distribution width, Hypertension, Kidney, Observational study, Prognosis, Kidney diseases, Risk factors, Prognostic markers, Biomarkers

## Abstract

Platelet distribution width (PDW), which represents the heterogeneity of platelet size, can predict a poor prognosis in various populations. However, the PDW-to-serum albumin ratio (PAR) has not been evaluated in any disease population, and whether the PAR could be a prognostic marker in hypertension remains unknown. The relationship between the PAR and adverse outcomes was examined retrospectively using longitudinal data of 1,578 patients with hypertension from the Fukushima Cohort Study. Participants were categorized into tertiles by baseline PAR. The primary endpoint of the present study was a kidney event, defined as a combination of a 50% decline in eGFR from baseline and end-stage kidney disease requiring kidney replacement therapy. During the median follow-up period of 5.4 years, 146 patients had kidney events. The higher PAR group (tertile 3) showed an increased incidence of kidney events on Kaplan–Meier curve analysis. Compared with the lowest PAR tertile, the highest PAR tertile (tertile 3) showed a significantly higher risk of kidney events (adjusted hazard ratio 3.74, 95% confidence interval (CI) 1.65–8.48). Similar relationships were observed for risks of all-cause death and cardiovascular events. The predictive value of the PAR for kidney events was superior to that of PDW alone. The areas under the curves for PDW and the PAR were 0.61 (95% CI 0.56–0.66) and 0.77 (95% CI 0.74–0.81), respectively (P < 0.001). The PAR could be a useful predictive marker of adverse outcomes in this population.

**Supplementary Information:**

The online version contains supplementary material available at 10.1038/s41598-025-05575-z.

##  Introduction

Hypertension, one of the major causes of atherosclerosis through vascular endothelial dysfunction and sustained activation of inflammatory factors, contributes to the progression of cerebrovascular and cardiovascular diseases, as well as deterioration in quality of life and a poor prognosis^1^. Chronic hypertension not only contributes to these atherosclerotic diseases, but also accelerates kidney disease progression and is one of the major causes of kidney failure.

The increasing number of kidney failure patients is a global public health problem, with Japan being one of the countries with the highest incidence of kidney failure. Regarding the causes of kidney failure, although diabetic nephropathy (38.3%) was the most common cause in incident chronic dialysis patients in Japan during 2023, its proportion has been decreasing in recent years. In contrast, the proportion of kidney failure caused by hypertensive nephropathy (19.3%) has been increasing steadily. Thus, early risk stratification and intensive management of patients with hypertension are essential for preventing kidney disease progression and reducing the number of incident dialysis patients, particularly in Japan.

Platelet distribution width (PDW), defined as the distribution width (in femtoliters, fL) at 20% of the total height of the platelet size distribution curve, is an indicator representing the heterogeneity of platelet size and one of the markers of platelet activity^2–5^. Higher levels of PDW are reportedly related to atherosclerosis, cardiovascular disease, cerebrovascular disease, and systemic inflammation^2,3,6,7^. Several recent studies showed that PDW has prognostic value for adverse outcomes including cardiovascular events, all-cause mortality, and cardiovascular mortality in patients with heart failure^8,9^ myocardial infarction^3,10^ pulmonary thromboembolism^11^deep venous thrombosis^12^ and pulmonary arterial hypertension^13,14^. PDW is also related to mortality in patients with carcinoma^15^ critically ill patients in the intensive care unit^16,17^ hospitalized patients on internal medicine wards^18^ and the general population^19^. Recently, several studies showed that higher PDW is associated with cardiovascular events and mortality in kidney failure patients on chronic hemodialysis^20^ and peritoneal dialysis^21–23^ whereas PDW is associated with a lower risk of cardiovascular events in chronic kidney disease (CKD) patients not on dialysis^24^. However, there has been no research on the interaction between PDW and adverse outcomes in patients with hypertension receiving care.

In contrast, red cell distribution width (RDW), a marker reflecting the degree of heterogeneity in erythrocyte volume, has been widely reported to be related to a poor prognosis in various disease populations^25–28^. Most recently, the RDW-to-serum albumin ratio (RAR), a combined index of RDW and serum albumin, has been identified as a potential marker for adverse clinical outcomes in various conditions^29,30^. Our recent report suggested that the discriminating ability of RAR is superior to that of RDW alone, particularly for kidney prognosis^31^. The PDW-to-serum albumin ratio (PAR) could also be a marker with better prognostic value for adverse kidney events than PDW alone, although the PAR has not been verified in any disease population.

Therefore, we first hypothesized that a higher PDW is associated with kidney disease progression and aimed to evaluate whether the prognostic value for kidney events improved when using the PAR compared with PDW alone in patients with hypertension receiving standard care using longitudinal data from the Fukushima Cohort Study.

## Materials and methods

### Study population

The Fukushima Cohort Study was a prospective survey of outpatients under the clinical care of specialists in nephrology or diabetology at Fukushima Medical University Hospital investigating the relationships between clinical characteristics and adverse outcomes, such as kidney events, kidney failure, cardiovascular events, and death. Outpatients having one or more cardiovascular risk factors, such as CKD, hypertension, diabetes mellitus, and dyslipidemia were included in the cohort. Detailed information on this cohort has been described elsewhere^32–35^. Total 2,724 participants were enrolled between June 2012 and July 2014. In the present study, participants who had hypertension with an estimated glomerular filtration rate (eGFR) ≥ 15 mL/min/1.73 m^2^ were included and investigated. Patients younger than 18 years old and without data on serum creatinine, PDW, and serum albumin were excluded. The study was registered in the University Hospital Medical Information Network Clinical Trials Registry (UMIN-CTR) under study ID UMIN000040848. The protocol was approved by the Ethics Committee of Fukushima Medical University (approval no. 2001), and the study was conducted in accordance with the Declaration of Helsinki. All patients provided written, informed consent to participate prior to enrolment.

### Data collection

Clinical information regarding demographics, comorbidities, medications, and blood examination results at baseline was obtained from the medical records of patients. Blood pressure was measured by trained staff using a standard sphygmomanometer or an automated device with the patient in a sitting position. Proteinuria was defined as positive dipstick result ≥1+. Serum creatinine was measured by an enzyme assay method, and eGFR was calculated using an estimation formula specifically designed for Japanese people^36^. Serum albumin, hemoglobin, platelet, PDW, and low-density lipoprotein cholesterol levels were measured according to the automated standardized laboratory technique of the clinical laboratory at our institution. Hypertension was defined as follows: (1) systolic blood pressure ≥ 140 mmHg; or (2) diastolic blood pressure ≥ 90 mmHg; or (3) use of antihypertensive medication. Diabetes mellitus was identified as follows: (1) fasting plasma glucose concentration ≥ 126 mg/dL; (2) hemoglobin A1c value (National Glycohemoglobin Standardization Program) ≥ 6.5%; or (3) use of insulin or oral antihyperglycemic drugs.

### Exposures and outcomes

The primary exposure of interest for the present study was the PAR calculated at baseline using the following formula: PAR = PDW (%)/serum albumin (g/dL). Patients were categorized into tertiles according to the baseline PAR. The primary endpoint of the present study was kidney events, defined as a combination of a 50% decline in eGFR from baseline and kidney failure requiring kidney replacement therapy. The secondary endpoints were all-cause death and cardiovascular events including fatal or non-fatal myocardial infarction, angina pectoris, sudden death, congestive or acute heart failure, arrhythmia, cerebrovascular disorder, chronic arteriosclerosis obliterans, and aortic disease. Information on renal replacement therapy and cardiovascular events was obtained from the medical records by attending physicians. Based on The Japanese Society of Hypertension Guidelines for the Management of Hypertension^37^ standard hypertension treatment was administered to all participants in the present study.

### Statistical analyses

Baseline characteristics of the study patients are expressed as percentages for categorical data and median and interquartile ranges for continuous variables with skewed distributions. The Kruskal–Wallis test and one-factor analysis of variance were used to compare median values, and Tukey’s test was used to evaluate differences in proportions. Kaplan–Meier curves and the log-rank test were used to compare event-free survivals of patients by PDW and PAR tertiles. Multivariable survival analyses using Cox proportional hazards models adjusted for confounding factors were used to examine associations between PDW and PAR tertiles at baseline and kidney events, all-cause death, and cardiovascular events. In addition to crude analyses, three multivariable models were created to adjust for covariates. Model 1 included age and sex, and Model 2 adjusted for Model 1 covariates plus smoking history, history of cardiovascular disease, diabetes mellitus, body mass index, systolic blood pressure, diastolic blood pressure, and eGFR. Model 3 adjusted for Model 2 covariates plus hemoglobin, platelet, LDL-cholesterol, proteinuria, use of angiotensin-converting enzyme (ACE) inhibitors or angiotensin II receptor blockers (ARBs), and use of antiplatelet agents. Results from the models are expressed as hazard ratios (HRs) with 95% confidence intervals (CIs) and *P* values based on the Wald chi-squared statistic. For sensitivity analysis, the relationships of PDW and the PAR to kidney events, all-cause death, and cardiovascular events were estimated using restricted cubic spline functions with four knots, at the 5th, 35th, 65th, and 95th percentiles of each index. The area under the curve (AUC) values of the receiver-operating characteristic (ROC) curves were examined to compare the predictive values of PDW and the PAR using the DeLong test. The predictive values of PDW and PAR were evaluated using Harrell’s C-statistic. Additionally, the incremental predictive value for kidney events was assessed by adding PAR to multivariable model 3, as described in the Cox regression analysis above. Further, subgroup analyses were performed to assess the relationships of PDW and PAR tertiles with kidney events, all-cause death, and cardiovascular events stratified by age (< 65 y and ≥ 65 y), sex distribution, diabetes mellitus, eGFR levels (≥ 45 mL/min/1.73 m^2^ and < 45 mL/min/1.73 m^2^), and proteinuria. All statistical analyses were performed using SPSS software (version 29; IBM Corporation, Chicago, IL, USA) and STATA MP, version 15.1 (Stata Corp, College Station, TX, USA). A significant difference was defined as *P* < 0.05.

## Results

### Patient characteristics

**Fig. 1:**
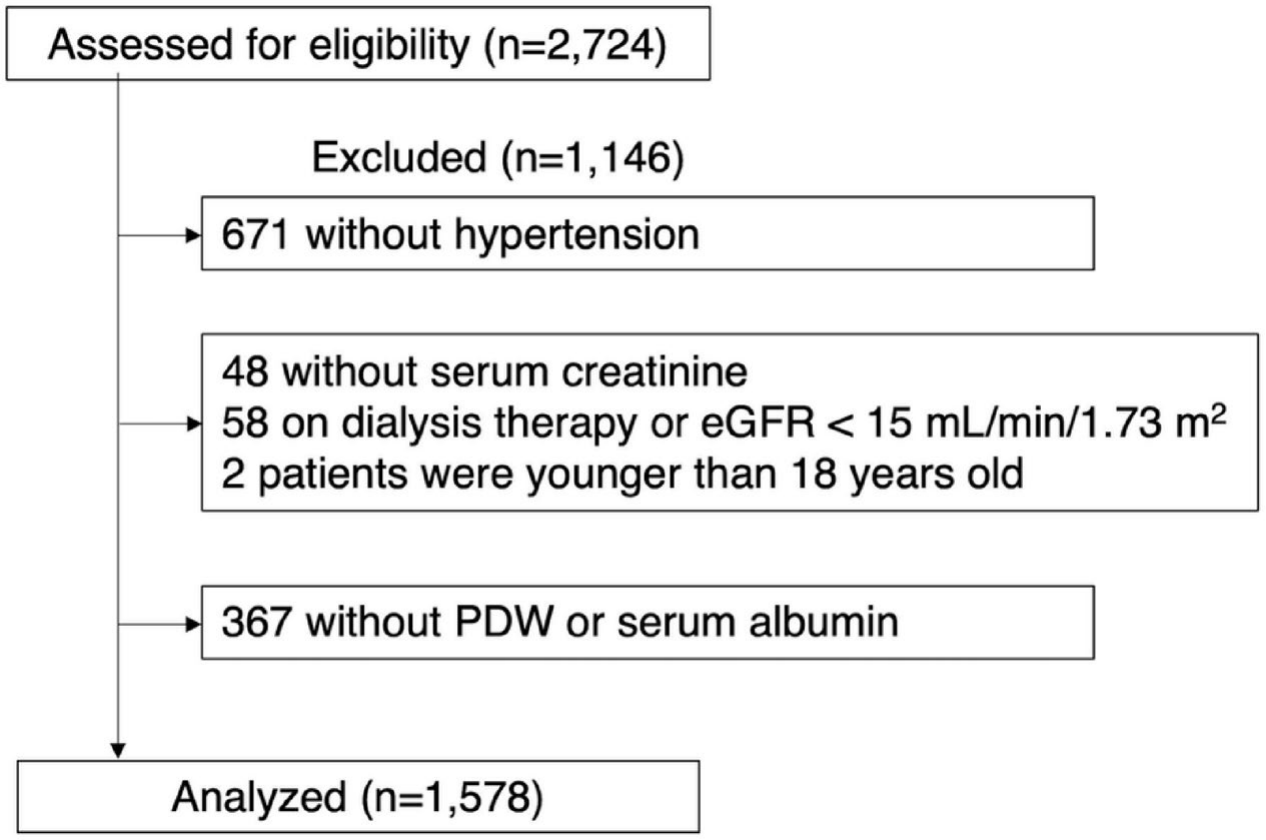
Flow of participants through the present study. eGFR, estimated glomerular filtration rate; PDW, platelet distribution width.

Figure 1 shows the flow of participants through the present study, and 1,578 of the 2,724 subjects enrolled in the Fukushima Cohort study were included in the present analyses. Table 1 shows the baseline characteristics by PDW tertile. Median age was 64.5 years, 54.3% were male, median eGFR was 62.9 mL/min/1.73 m^2^and median PDW was 16.7%. Participants in the highest PDW tertile (tertile 3) were older and more likely to have a smoking history, cardiovascular disease history, diabetes mellitus, proteinuria, antiplatelet agent use, and anticoagulant medication use. Compared with those in lower PDW tertiles, diastolic blood pressure, eGFR, serum albumin, hemoglobin, and platelet counts were lower in these participants. Baseline characteristics by the PAR are shown in Table 2. Similar results were also found for the PAR and baseline characteristics, in addition to the results above, participants in the highest PAR tertile had lower body mass index and lower LDL-cholesterol than those in the lower PAR tertile. At baseline, sodium-glucose co-transporter 2 inhibitors were not yet approved or available for clinical use in Japan, and thus none of the participants were receiving them.

**Table 1:** Baseline characteristics of patients by PDW tertile.

Variable	Missing data		Total	PDW			*P* for trend
	*n*	%		Tertile 1	Tertile 2	Tertile 3	
Age (y)	0	0	64.5 (56.0–74.0)	63.0 (52.0–72.0)	65.0 (57.0–73.0)	66.0 (58.0–75.0)	< 0.001
Male (%)	0	0	54.3	41.7	54.0	65.0	< 0.001
Body mass index (kg/m^2^)	0	0	24.6 (22.3–27.5)	24.5 (21.9–27.7)	24.6 (22.5–27.4)	24.6 (22.2–27.5)	0.723
Smoking history (%)	67	0.7	47.6	37.4	45.8	57.7	< 0.001
History of cardiovascular disease (%)	0	0	11.3	7.1	10.5	15.5	< 0.001
Diabetes mellitus (%)	0	0	50.2	38.3	47.6	62.5	< 0.001
Systolic blood pressure (mmHg)	0	0	134 (122–146)	134 (121–146)	133 (122–145)	135 (123–148)	0.456
Diastolic blood pressure (mmHg)	0	0	77 (69–86)	79 (70–87)	76 (69–85)	77 (68–85)	0.011
eGFR (mL/min/1.73 m^2^)	0	0	62.9 (47.5–77.0)	66.7 (52.7–78.7)	63.5 (50.2–77.0)	57.7 (42.7–74.5)	< 0.001
Serum albumin (g/dL)	0	0	4.0 (3.8–4.2)	4.0 (3.8–4.3)	4.1 (3.8–4.3)	4.0 (3.7–4.2)	0.006
Hemoglobin (g/dL)	9	0.1	13.2 (12.1–14.3)	13.2 (12.1–14.2)	13.3 (12.3–14.4)	13.1 (11.8–14.2)	0.027
Platelets (/µL)	10	0.1	20.4 (17.1–24.4)	23.4 (19.9–27.6)	20.3 (17.5–23.9)	18.3 (14.6–21.7)	< 0.001
PDW (%)	0	0	16.7 (16.4–17.1)	16.2 (16.1–16.4)	16.7 (16.6–16.8)	17.3 (17.1–17.6)	< 0.001
PAR	0	0	4.18 (3.93–4.47)	4.00 (3.79–4.29)	4.12 (3.93–4.35)	4.35 (4.12–4.68)	< 0.001
LDL cholesterol (mg/dL)	124	7.9	104 (85–123)	106 (89–125)	105 (83–123)	102 (84–124)	0.219
Proteinuria (dipstick) (%)	58	3.7					0.013
(−)/(±)			72.8	74.2	76.0	68.6	
(1+)			12.7	10.8	12.7	14.0	
(2+)			7.4	6.9	6.4	8.7	
(3+ ≤)			3.4	3.2	1.8	5.2	
ACE inhibitor or ARB (%)	0	0	74.3	73.5	75.6	73.5	0.663
Antiplatelet agent (%)	0	0	15.4	11.6	14.9	19.0	0.004
Anticoagulant (%)	0	0	6.8	4.7	5.8	9.6	0.004

The values in the table indicate medians (25-75th percentiles) or percentages, as appropriate. PDW, platelet distribution width; PAR, PDW-to-albumin ratio; eGFR, estimated glomerular filtration rate; LDL, low-density lipoprotein; ACE, angiotensin-converting enzyme; ARB, angiotensin II receptor blocker.

**Table 2:** Baseline characteristics of patients by PAR tertile.

Variable	PAR			*P* for trend
	Tertile 1	Tertile 2	Tertile 3	
Age (y)	62.0 (51.0–68.0)	66.0 (59.0–74.0)	68.0 (59.0–77.0)	< 0.001
Male (%)	51.8	54.9	56.0	0.376
Body mass index (kg/m^2^)	25.1 (22.5–28.0)	24.6 (22.2–27.4)	24.2 (22.0–27.2)	0.024
Smoking history (%)	46.1	48.9	53.8	0.050
History of cardiovascular disease (%)	7.4	12.3	13.8	0.003
Diabetes mellitus (%)	36.9	53.4	59.1	< 0.001
Systolic blood pressure (mmHg)	135 (121–147)	133 (123–145)	133 (121–147)	0.503
Diastolic blood pressure (mmHg)	80 (72–88)	77 (69–85)	75 (67–83)	< 0.001
eGFR (mL/min/1.73 m^2^)	69.6 (58.0–83.1)	62.2 (49.1–75.1)	54.1 (38.8–71.3)	< 0.001
Serum albumin (g/dL)	4.3 (4.2–4.5)	4.0 (3.9–4.1)	3.6 (3.4–3.8)	< 0.001
Hemoglobin (g/dL)	13.8 (13.0–14.8)	13.3 (12.1–14.4)	12.3 (11.3–13.4)	< 0.001
Platelets (/µL)	22.0 (19.0–25.8)	19.8 (16.5–23.2)	19.6 (15.5–24.1)	< 0.001
PDW (%)	16.5 (16.3–16.8)	16.8 (16.4–17.1)	17.0 (16.6–17.3)	< 0.001
PAR	3.84 (3.69–3.93)	4.15 (4.08–4.24)	4.65 (4.46–4.97)	< 0.001
LDL-cholesterol (mg/dL)	109 (91–128)	105 (88–123)	98 (78–118)	< 0.001
Proteinuria (dipstick) (%)				< 0.001
(−)/(±)	82.3	76.5	60.4	
(1+)	9.2	12.3	16.4	
(2+)	4.0	5.6	12.2	
(3+ ≤)	0.4	1.7	7.9	
ACE inhibitor or ARB (%)	73.1	74.9	74.8	0.767
Antiplatelet agent (%)	10.0	16.4	19.3	< 0.001
Anticoagulant (%)	2.0	6.9	11.2	< 0.001

The values in the table indicate medians (25-75th percentiles) or percentages, as appropriate. PAR, PDW-to-albumin ratio; eGFR, estimated glomerular filtration rate; PDW, platelet distribution width; LDL, low-density lipoprotein; ACE, angiotensin-converting enzyme; ARB, angiotensin II receptor blocker.

### Kidney events

**Fig. 2:**
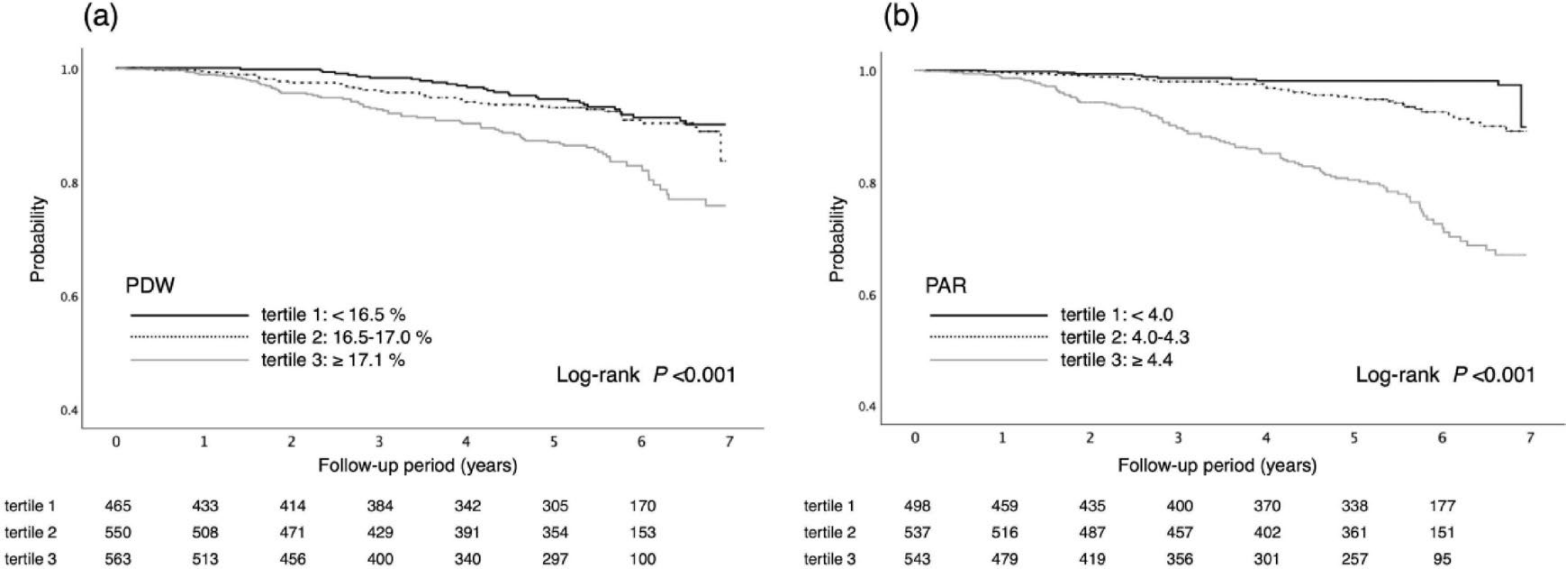
Kaplan-Meier curves for the incidence of kidney events by PDW (**a**) and PAR (**b**) tertiles at baseline in patients with hypertension. PDW, platelet distribution width; PAR, PDW-to-albumin ratio.

**Fig. 3:**
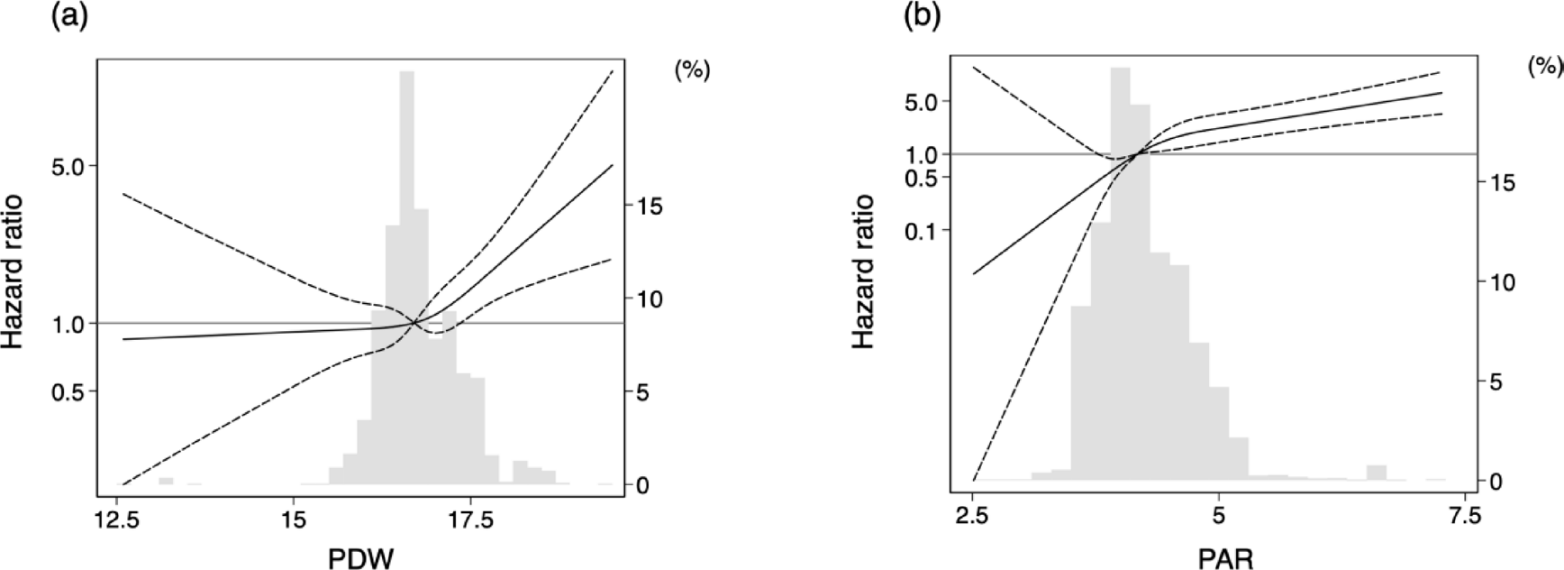
Distributions and model-adjusted restricted cubic splines assessing the relationship of PDW (**a**) and PAR (**b**) to kidney events. The solid lines represent adjusted hazard ratio estimates, and the dashed lines represent 95% confidence intervals, respectively. Model adjusted for age, sex, smoking history, history of cardiovascular disease, diabetes mellitus, body mass index, systolic blood pressure, diastolic blood pressure, eGFR, hemoglobin, platelet, LDL-cholesterol, proteinuria, use of ACE inhibitor or ARB, and use of antiplatelet agent. PDW, platelet distribution width; PAR, PDW-to-albumin ratio; eGFR, estimated glomerular filtration rate; LDL, low-density lipoprotein; ACE, angiotensin-converting enzyme; ARB, angiotensin II receptor blocker.

**Fig. 4:**
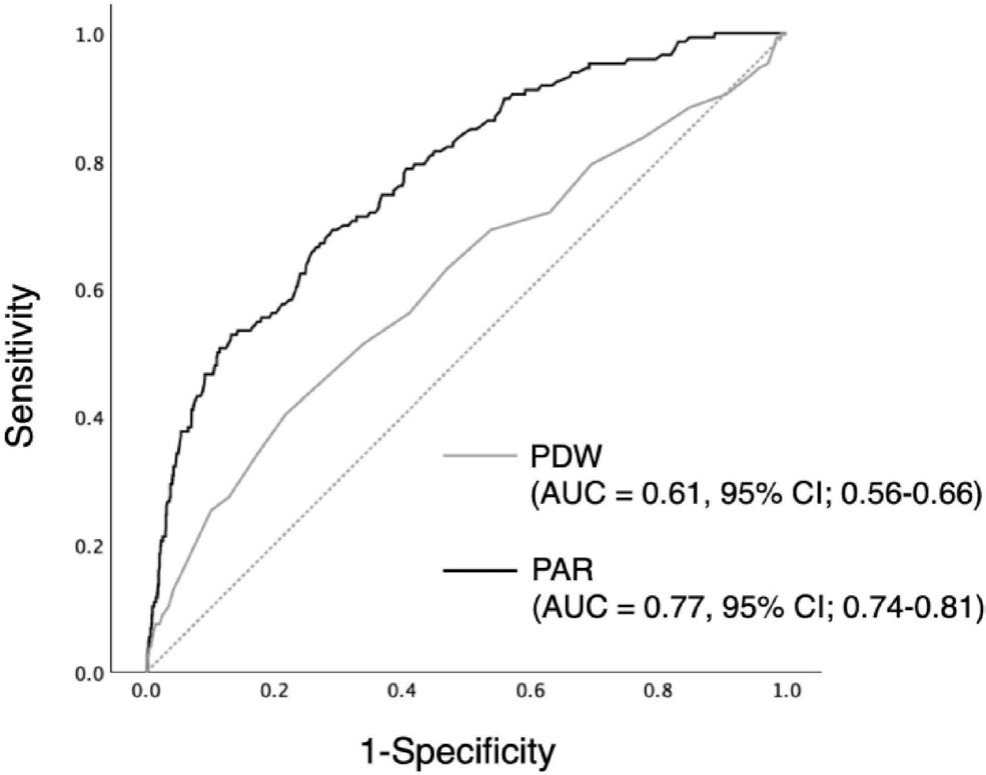
Comparison of the ROC curves of PDW and PAR for predicting kidney events in patients with hypertension. The AUC for PAR was significantly higher than the AUC for PDW (***P*** < 0.001). PDW, platelet distribution width; PAR, PDW-to-albumin ratio; AUC, area under the curve; CI, confidence interval; ROC, receiver-operating characteristic.

During the median observational period of 5.4 years, 146 of 1,578 patients had kidney events. Significantly higher incidences of kidney events were observed in participants in the higher tertiles of PDW and PAR, respectively (*P* < 0.001, Fig. 2). Compared with the lowest PDW tertile (tertile 1, as a reference value), participants in the highest tertile (tertile 3) showed a significantly higher risk of kidney events on crude Cox regression analysis. These associations remained significant after adjustment in multivariable models (Table 3). When PDW levels were treated as a continuous variable, a 1-increase in PDW levels was associated with a 57% increase (95% confidence interval (CI) 1.18–2.10) in the risk of kidney events (Model 3). Similar results were also observed between the PAR and kidney events. A 1-increase in PAR levels was associated with an 87% increase (95% CI 1.52–2.29) in the risk of kidney events (Model 3). The restricted cubic spline curves showed that increasing PDW, as well as the PAR, was associated with a higher risk of kidney events (Fig. 3). On ROC curve analysis, the predictive value of the PAR for kidney events was superior to that of PDW (Fig. 4). The AUCs for PDW and the PAR were 0.61 (95% CI 0.56–0.66) and 0.77 (95% CI 0.74–0.81), respectively (*P* < 0.001). The predictive values of PDW and the PAR were also investigated using Harrell’s C-statistic, and similar results were observed. The C-statistics for PDW and the PAR were 0.63 (95% CI 0.58–0.68) and 0.80 (95% CI 0.76–0.84), respectively (*P* < 0.001, Table 4). The incremental predictive value for kidney events was also assessed when the PAR was added to Model 3 in the Cox regression analysis, which included age, sex, smoking history, history of cardiovascular disease, diabetes mellitus, body mass index, systolic blood pressure, diastolic blood pressure, eGFR, hemoglobin, platelets, LDL-cholesterol, proteinuria, use of ACE inhibitor or ARB, and use of antiplatelet agents. The C-statistic for kidney events were significantly increased with the addition of the PAR to Model 3. The C-statistics for Model 3 and Model 3 plus the PAR were 0.85 (95% CI 0.81–0.89) and 0.87 (95% CI 0.84–0.91), respectively (*P* = 0.011, Table 4).

**Table 3:** Hazard ratios and 95% confidence intervals for the associations of PDW and the PAR ratio with kidney events.

	Tertile 1	Tertile 2	Tertile 3	Per 1-increase
PDW	Hazard ratio (95% confidence interval)			
Crude	1.00 (ref.)	1.24 (0.77–1.98)	2.58 (1.69–3.95)	2.18 (1.68–2.83)
Model 1	1.00 (ref.)	1.18 (0.73–1.89)	2.35 (1.53–3.62)	2.08 (1.59–2.72)
Model 2	1.00 (ref.)	1.11 (0.67–1.83)	1.59 (1.00–2.52)	1.63 (1.23–2.15)
Model 3	1.00 (ref.)	1.14 (0.66–1.98)	1.73 (1.03–2.88)	1.57 (1.18–2.10)
Number of participants	465	550	563	
Number of events	30	41	75	
Incidence rates (/1,000 person-years)	13.0	15.6	31.0	
PAR	Hazard ratio (95% confidence interval)			
Crude	1.00 (ref.)	3.21 (1.59–6.50)	12.6 (6.55–24.1)	2.61 (2.33–2.91)
Model 1	1.00 (ref.)	3.13 (1.54–6.35)	12.3 (6.39–23.6)	2.55 (2.28–2.86)
Model 2	1.00 (ref.)	2.23 (1.06–4.69)	5.95 (2.94–12.0)	2.34 (2.04–2.69)
Model 3	1.00 (ref.)	1.86 (0.80–4.33)	3.74 (1.65–8.48)	1.87 (1.52–2.29)
Number of participants	498	537	543	
Number of events	10	34	102	
Incidence rates (/1,000 person-years)	4.1	12.7	46.2	

Model 1, adjusted for age and sex. Model 2, adjusted for Model 1 covariates plus smoking history, history of cardiovascular disease, diabetes mellitus, body mass index, systolic blood pressure, diastolic blood pressure, and eGFR. Model 3, adjusted for Model 2 covariates plus hemoglobin, platelets, LDL-cholesterol, proteinuria, use of ACE inhibitor or ARB, and use of antiplatelet agents. PDW, platelet distribution width; PAR, PDW-to-albumin ratio; eGFR, estimated glomerular filtration rate; LDL, low-density lipoprotein; ACE, angiotensin-converting enzyme; ARB, angiotensin II receptor blocker.

**Table 4:** Comparison of the predictive values for kidney events using harrell’s C-statistic.

	C-statistic (95% confidence interval)	*P*
		< 0.001
PDW	0.63 (0.58–0.68)	
PAR	0.80 (0.76–0.84)	
		0.011
Model 3	0.85 (0.81–0.89)	
Model 3 plus PAR	0.87 (0.84–0.91)	

Model 3, including age, sex, smoking history, history of cardiovascular disease, diabetes mellitus, body mass index, systolic blood pressure, diastolic blood pressure, eGFR, hemoglobin, platelets, LDL-cholesterol, proteinuria, use of ACE inhibitor or ARB, and use of antiplatelet agents. PDW, platelet distribution width; PAR, PDW-to-albumin ratio; eGFR, estimated glomerular filtration rate; LDL, low-density lipoprotein; ACE, angiotensin-converting enzyme; ARB, angiotensin II receptor blocker.

## Subgroup analyses

Subgroup analyses were conducted to evaluate the associations of platelet distribution width (PDW) and the platelet-to-albumin ratio (PAR) with kidney events. These analyses revealed no significant effect modification by baseline covariates, including age (< 65 and ≥ 65 y), sex, diabetes mellitus, estimated glomerular filtration rate (eGFR ≥ 45 and < 45 mL/min/1.73 m²), serum albumin levels (< 4.0 and ≥ 4.0 g/dL), and proteinuria (Tables 5 and 6). The interaction terms between PDW or PAR and these covariates were not statistically significant. Although a higher PDW was significantly associated with kidney events only in participants with serum albumin < 4.0 g/dL and those without proteinuria (Table 5), a higher PAR was consistently associated with an increased risk of kidney events across all subgroups (Table 6).

### All-cause death and cardiovascular events

During the observational period, 116 of 1,578 patients died, and 169 experienced cardiovascular events. On Kaplan–Meier analyses, significantly higher incidences of all-cause death and cardiovascular events were found in participants in higher tertiles of PDW and PAR, respectively (*P* < 0.001, Supplementary Figure S1, S2). Compared with the lowest PDW tertile (tertile 1, as a reference value), participants in the highest tertile (tertile 3) showed a significant and independently higher risk of all-cause death (**Supplementary Table ****S1**). Similar results were also observed between the PAR and all-cause death. Regarding cardiovascular events, a higher PAR was significantly associated with the risk of cardiovascular events on multivariable Cox regression analysis. Although a higher PDW was associated with the risk of cardiovascular events in the univariate model, significant associations were attenuated and disappeared in the multivariable model (Supplementary Table S2). The restricted cubic spline curves showed that increasing PDW, as well as PAR, was associated with a higher risk of all-cause death and cardiovascular events (**Supplementary **Figure S3, S4). On ROC curve analysis, the predictive values of the PAR were non-superior to those of PDW for both all-cause death (*P* = 0.066) and cardiovascular events (*P* = 0.152) (Supplementary Figure S5). According to Harrell’s C-statistic, the predictive value of the PAR was superior to that of PDW for all-cause mortality, but not for cardiovascular events. The C-statistics for all-cause mortality were 0.63 (95% CI 0.57–0.68) for PDW and 0.69 (95% CI 0.65–0.74) for the PAR (*P* = 0.040). For cardiovascular events, the C-statistics were 0.62 (95% CI 0.58–0.67) for PDW and 0.66 (95% CI 0.61–0.70) for the PAR (*P* = 0.186).

**Table 5:** Subgroup analyses of the association of PDW level with kidney events.

		Hazard ratio (95% confidence interval)			*P* for interaction
		Tertile 1	Tertile 2	Tertile 3	
Overall		1.00 (ref.)	1.08 (0.67–1.76)	1.64 (1.05–2.55)	
Age (y)					
≥ 65 y		1.00 (ref.)	1.02 (0.51–2.05)	1.42 (0.74–2.70)	0.764
< 65 y		1.00 (ref.)	1.07 (0.54–2.11)	1.81 (0.98–3.33)	
Sex distribution					
Male		1.00 (ref.)	0.97 (0.51–1.85)	1.51 (0.84–2.72)	0.709
Female		1.00 (ref.)	1.18 (0.56–2.48)	1.83 (0.94–3.59)	
Diabetes mellitus					
Yes		1.00 (ref.)	0.89 (0.45–1.78)	1.61 (0.90–2.86)	0.738
No		1.00 (ref.)	1.36 (0.67–2.75)	1.60 (0.80–3.20)	
eGFR (mL/min/1.73 m^2^)					
≥ 45		1.00 (ref.)	1.88 (0.80–4.42)	2.28 (0.98–5.32)	0.124
< 45		1.00 (ref.)	0.70 (0.38–1.29)	1.31 (0.77–2.23)	
Serum albumin (g/dL)					
< 4.0		1.00 (ref.)	1.17 (0.66–2.06)	1.85 (1.13–3.04)	0.616
≥ 4.0		1.00 (ref.)	1.48 (0.55–4.01)	1.79 (0.67–4.77)	
Proteinuria					
Positive		1.00 (ref.)	0.92 (0.50–1.68)	1.38 (0.82–2.32)	0.463
Negative		1.00 (ref.)	1.77 (0.73–4.34)	2.64 (1.11–6.30)	

Adjusted for age, sex, diabetes mellitus, systolic blood pressure, eGFR, and proteinuria. PDW, platelet distribution width; eGFR, estimated glomerular filtration rate.

**Table 6:** Subgroup analyses of the association of PAR level with kidney events.

		Hazard ratio (95% confidence interval)			*P* for interaction
		Tertile 1	Tertile 2	Tertile 3	
Overall		1.00 (ref.)	1.62 (0.79–3.33)	4.33 (2.20–8.51)	
Age (y)					
≥ 65 y		1.00 (ref.)	1.26 (0.45–3.52)	4.00 (1.54–10.4)	0.551
< 65 y		1.00 (ref.)	2.12 (0.77–5.83)	4.74 (1.80–12.5)	
Sex distribution					
Male		1.00 (ref.)	1.15 (0.50–2.66)	3.18 (1.46–6.92)	0.219
Female		1.00 (ref.)	3.63 (0.80–16.4)	9.60 (2.24–41.1)	
Diabetes mellitus					
Yes		1.00 (ref.)	1.93 (0.55–6.79)	7.45 (2.29–24.3)	0.362
No		1.00 (ref.)	1.49 (0.62–3.61)	2.64 (1.13–6.17)	
eGFR (mL/min/1.73 m^2^)					
≥ 45		1.00 (ref.)	1.59 (0.53–4.79)	6.63 (2.49–17.7)	0.528
< 45		1.00 (ref.)	1.19 (0.45–3.17)	2.87 (1.14–7.26)	
Proteinuria					
Positive		1.00 (ref.)	1.05 (0.39–2.85)	2.97 (1.18–7.46)	0.786
Negative		1.00 (ref.)	2.28 (0.81–6.39)	6.03 (2.23–16.3)	

Adjusted for age, sex, diabetes mellitus, systolic blood pressure, eGFR, and proteinuria. PAR, PDW-to-albumin ratio; eGFR, estimated glomerular filtration rate.

## Discussion

In this retrospective, cohort study, the PAR, a combined index of PDW and serum albumin, was assessed to determine its independent effects on clinical adverse outcomes in patients with hypertension, which have not been investigated sufficiently. Higher PAR levels were found to be significantly and independently associated with an increased risk of adverse clinical outcomes, including all-cause mortality, cardiovascular events, and kidney events defined as the combination of a 50% decline in eGFR from baseline and kidney failure requiring kidney replacement therapy in Japanese patients with hypertension receiving standard treatment based on The Japanese Society of Hypertension Guidelines. Furthermore, the PAR demonstrated a superior predictive value for kidney events than PDW alone in this population. Subgroup analyses also showed that the PAR had significant effects on kidney disease progression across various patient subgroups, and it was identified as a useful and reliable marker for clinical risk management in hypertension care.

Platelets play a critical role in the processes of blood coagulation, inflammation, and the immune response^38–40^. Platelet activation is an important reaction in hemostasis, since it helps prevent excessive bleeding^41^but it might also contribute to arteriosclerotic disease progression through arterial thrombosis^42^. PDW, one of the platelet indices, is thought to be a marker of platelet activity, and an increased PDW reflects platelet activation^2–5,20,24^. Associations between higher PDW levels and a poor prognosis have been reported in various clinical studies across different conditions. A higher PDW has been reportedly associated with the risk of mortality and/or cardiovascular events in patients with heart failure^9,24^myocardial infarction^3,10^pulmonary arterial hypertesion^13,14^pulmonary embolism^11^deep venous thrombosis^12^cancer^15^critically ill patients in the intensive care unit^16,17^hospitalized patients on internal medicine wards^18^and the general population^19^. Regarding the kidney disease population, previous studies have reported inconsistent results. Ruiyan et al. found significant associations between increased PDW levels and a higher risk of all-cause mortality and cardiovascular mortality in patients on chronic hemodialysis^20^. In patients on peritoneal dialysis, increased PDW levels were associated with a higher risk of cardiovascular events^22^ and all-cause mortality and cardiovascular mortality^21,23^. However, Peng et al. reported that PDW levels had no significant effect on the risk of cardiovascular mortality in incident peritoneal dialysis patients^43^. In a recent study involving patients with non-dialysis-dependent CKD, the PDW level showed a marginal but significant association with a lower risk of cardiovascular events (adjusted HR 0.936, 95% CI 0.878–0.998, *P* = 0.044)^24^. These discrepancies might arise from the fact that the effect of a higher PDW, which reflects platelet activation, on disease progression differs across subject populations. Although a previous report suggested that patients with hypertension had a higher PDW than those without hypertension^44^associations between the PDW level and adverse outcomes have not yet been investigated in patients with hypertension receiving care. Thus, the present study is the first to confirm the significant and independent effects of an elevated PAR, as well as PDW, on kidney disease progression, all-cause mortality, and cardiovascular events, in a large cohort of hypertensive patients.

The present study also demonstrated that the prognostic value for kidney events was significantly improved by using the PAR than using PDW alone. Although the precise mechanism through which the PAR is related to adverse outcomes, including kidney disease progression, remains unclear, some potential mechanisms other than thrombosis formation, including inflammatory responses, malnutrition, and endothelial dysfunction, can also be considered. Chronic inflammation, to which higher platelet activity contributes, is reportedly associated with elevated PDW^45,46^ and leads to excessive proliferation of vascular smooth muscle cells and endothelial dysfunction, which could increase the risk of developing organ damage, including the kidneys^47^. Decreased serum albumin, considered as an important marker reflecting lower nutritional status and higher inflammatory status^48^was reported to be associated with a higher risk of a poor kidney prognosis in patients with CKD^49^. Therefore, the present results suggested that the PAR, which simultaneously reflects various conditions including platelet activity, inflammation, malnutrition, and endothelial dysfunction, could be a better marker for kidney prognosis in patients with hypertension receiving care.

The strength of the present study lies in its use of a large population database on standard hypertension treatment, along with the application of suitable statistical methods to ensure reliable results. However, several limitations of the present study should be noted. First, due to the nature of observational studies, the associations observed may not establish causal relationships between PDW or the PAR and kidney events, all-cause mortality, or cardiovascular events. Second, PDW and the PAR were only measured at baseline in this study, and changes during the follow-up period were not evaluated. The single assessment of PDW or the PAR might have led to potential misclassification in this population. Third, cardiovascular disease and dialysis outcomes were obtained directly from electronic medical records without formal adjudication. This approach may have introduced some degree of misclassification or variability in outcome ascertainment, which we acknowledge as a limitation of the study, as it may affect the precision of our results. Fourth, detailed information on the etiology of kidney disease progression was not available in the present study, and data on the degree of hematuria were also lacking. These limitations should be considered when interpreting the association between elevated PAR and kidney events in this population, as they may impact the understanding of the underlying causes and mechanisms.

In conclusion, this analysis provided longitudinal evidence of the associations between the PAR, as well as PDW, and adverse clinical outcomes in hypertensive patients receiving standard care. The present findings also indicated that the PAR had a superior prognostic value for kidney outcomes than PDW alone. The PAR, which can be easily calculated using PDW and serum albumin from routine laboratory tests, could serve as a valuable marker for risk stratification in clinical hypertension management.

## Electronic supplementary material

Below is the link to the electronic supplementary material.

Supplementary Material 1

Supplementary Material 2

## Data Availability

The datasets generated and/or analyzed during the present study are available from the corresponding author on reasonable request.
