# Supplementary material for: The relationship of the ratio of platelet distribution width to serum albumin with kidney disease progression in patients with hypertension
Source: Sci Rep. 2025 Jul 1;15:20480. doi: 10.1038/s41598-025-05575-z (PMC12217693; doi:10.1038/s41598-025-05575-z)
Supplement: Supplementary file 1 — Supplementary Material 1 [file 41598_2025_5575_MOESM1_ESM.pdf]

Table S1. Hazard ratios and 95% confidence intervals for the associations of PDW and the PAR with all-cause death

	Tertile 1	Tertile 2	Tertile 3	Per 1-increase
PDW	Hazard ratio (95% confidence interval)			
Crude	1.00 (ref.)	1.23 (0.71-2.13)	2.70 (1.66-4.39)	2.03 (1.53-2.70)
Model 1	1.00 (ref.)	1.07 (0.62-1.86)	2.10 (1.27-3.45)	1.75 (1.29-2.37)
Model 2	1.00 (ref.)	1.04 (0.59-1.85)	1.85 (1.10-3.11)	1.61 (1.16-2.22)
Number of events	22	31	63	
Incidence rates (/1,000 person-years)	9.0	11.1	24.3	
PAR	Hazard ratio (95% confidence interval)			
Crude	1.00 (ref.)	2.98 (1.60-5.54)	4.97 (2.73-9.05)	1.88 (1.64-2.14)
Model 1	1.00 (ref.)	2.39 (1.28-4.46)	3.63 (1.98-6.67)	1.91 (1.65-2.22)
Model 2	1.00 (ref.)	2.11 (1.10-4.04)	2.70 (1.41-5.18)	1.80 (1.52-2.13)
Number of events	13	42	61	
Incidence rates (/1,000 person-years)	5.0	15.0	24.9	

Model 1, adjusted for age and sex. Model 2, adjusted for Model 1 covariates plus smoking history, history of cardiovascular disease, diabetes mellitus, body mass index, systolic blood pressure, diastolic blood pressure, and eGFR. PDW, platelet distribution width; PAR, PDW-to-albumin ratio; eGFR, estimated glomerular filtration rate.

Table S2. Hazard ratios and 95% confidence intervals for the associations of PDW and the PAR with cardiovascular events

	Tertile 1	Tertile 2	Tertile 3	Per 1-increase
PDW	Hazard ratio (95% confidence interval)			
Crude	1.00 (ref.)	1.22 (0.78-1.89)	2.54 (1.71-3.78)	1.54 (1.20-1.97)
Model 1	1.00 (ref.)	1.08 (0.69-1.68)	2.04 (1.36-3.05)	1.33 (1.04-1.71)
Model 2	1.00 (ref.)	0.97 (0.61-1.54)	1.57 (1.02-2.42)	1.16 (0.92-1.46)
Model 3	1.00 (ref.)	0.86 (0.53-1.41)	1.44 (0.92-2.27)	1.12 (0.90-1.39)
Number of events	34	47	88	
Incidence rates (/1,000 person-years)	14.3	17.4	36.4	
PAR	Hazard ratio (95% confidence interval)			
Crude	1.00 (ref.)	2.44 (1.51-3.95)	4.19 (2.65-6.64)	1.62 (1.40-1.89)
Model 1	1.00 (ref.)	2.07 (1.28-3.36)	3.41 (2.14-5.43)	1.61 (1.36-1.91)
Model 2	1.00 (ref.)	1.61 (0.98-2.63)	2.11 (1.29-3.45)	1.40 (1.14-1.72)
Model 3	1.00 (ref.)	1.60 (0.95-2.71)	1.97 (1.15-3.39)	1.30 (1.01-1.66)
Number of events	23	60	86	
Incidence rates (/1,000 person-years)	9.1	22.4	37.8	

Model 1, adjusted for age and sex. Model 2, adjusted for Model 1 covariates plus smoking history, history of cardiovascular disease, diabetes mellitus, body mass index, systolic blood pressure, diastolic blood pressure, and eGFR. Model 3, adjusted for Model 2 covariates plus hemoglobin, platelets, LDL-cholesterol, proteinuria, use of ACE inhibitor or ARB, and use of antiplatelet agents. PDW, platelet distribution width; PAR, PDW-to-albumin ratio; eGFR, estimated glomerular filtration rate; LDL, low-density lipoprotein; ACE, angiotensin-converting enzyme; ARB, angiotensin II receptor blocker.
