# Supplementary material for: The relationship of the ratio of platelet distribution width to serum albumin with kidney disease progression in patients with hypertension
Source: Sci Rep. 2025 Jul 1;15:20480. doi: 10.1038/s41598-025-05575-z (PMC12217693; doi:10.1038/s41598-025-05575-z)
Supplement: Supplementary file 2 — Supplementary Material 2 [file 41598_2025_5575_MOESM2_ESM.pdf]

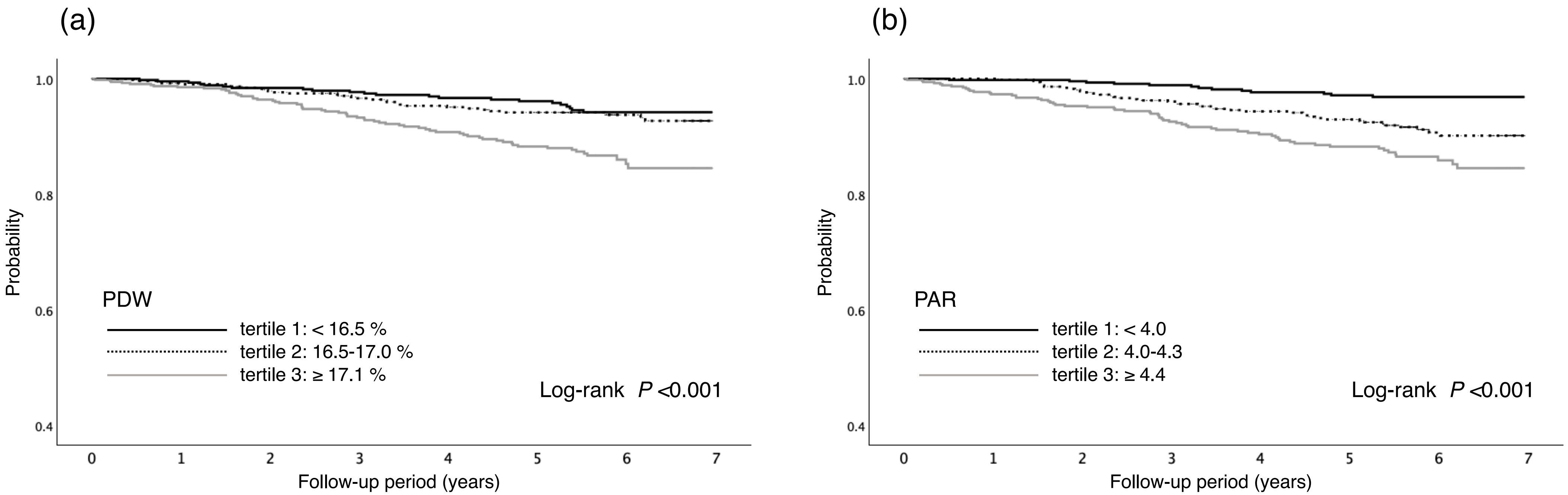

tertile 1	465	440	423	401	362	336	197
tertile 2	550	514	489	452	424	392	186
tertile 3	563	525	480	433	377	331	116

tertile 1	498	467	446	418	390	361	197
tertile 2	537	520	496	469	421	387	169
tertile 3	543	492	450	399	352	311	133

Figure S1. Kaplan-Meier curves for the incidence of all-cause death by PDW (a) and PAR (b) tertiles at baseline in patients with hypertension. PDW, platelet distribution width; PAR, PDW-to-albumin ratio.

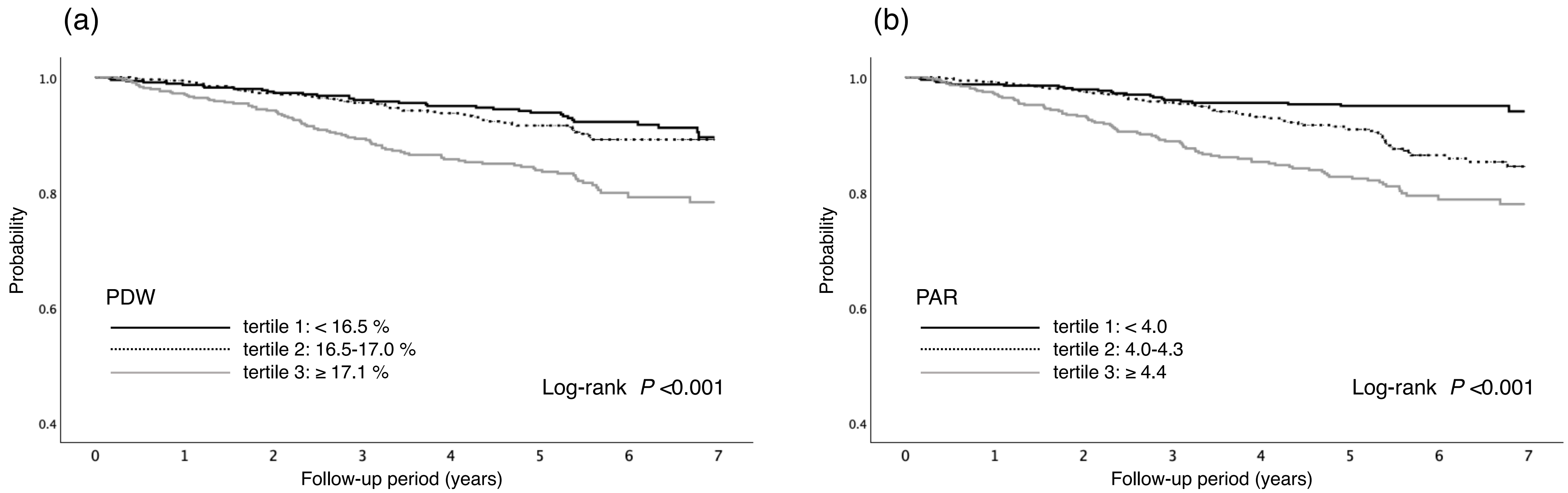

tertile 1	465	435	414	387	351	321	187
tertile 2	550	512	476	434	402	368	171
tertile 3	563	509	458	396	335	293	105

tertile 1	498	464	439	405	379	350	191
tertile 2	537	513	487	451	397	361	152
tertile 3	543	478	422	361	312	271	120

Figure S2. Kaplan-Meier curves for the incidence of cardiovascular disease by PDW (a) and PAR (b) tertiles at baseline in patients with hypertension. PDW, platelet distribution width; PAR, PDW-to-albumin ratio.

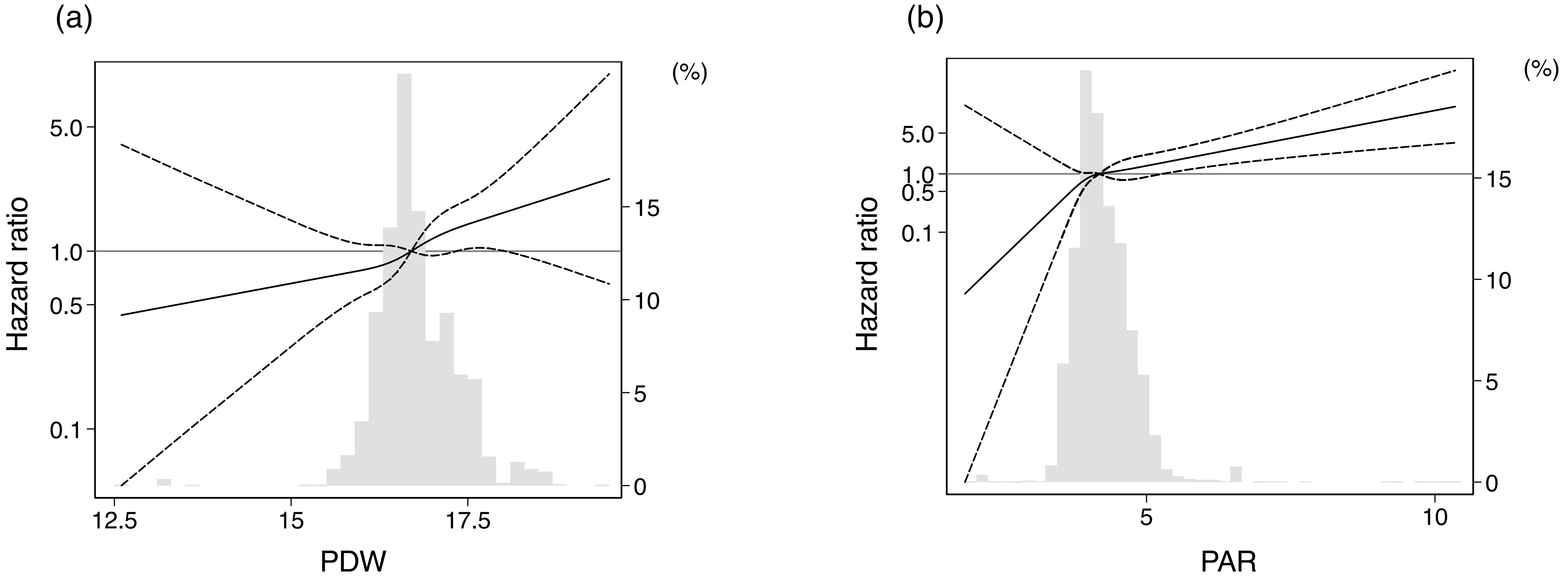

Figure S3. Distributions and model-adjusted restricted cubic splines assessing the relationship of PDW (a) and PAR (b) to all-cause death. The solid lines represent adjusted hazard ratio estimates, and the dashed lines represent 95% confidence intervals, respectively. Model adjusted for age, sex, smoking history, history of cardiovascular disease, diabetes mellitus, body mass index, systolic blood pressure, diastolic blood pressure, and eGFR. PDW, platelet distribution width; PAR, PDW-to-albumin ratio; eGFR, estimated glomerular filtration rate.

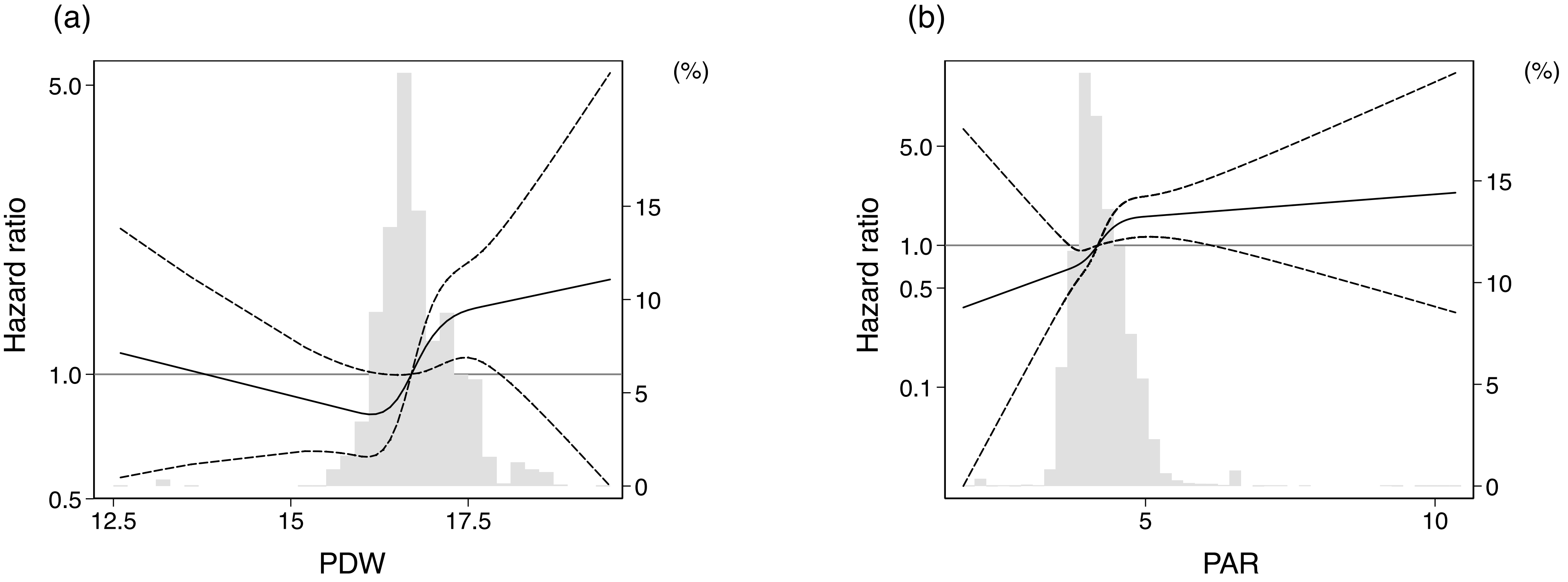

Figure S4. Distributions and model-adjusted restricted cubic splines assessing the relationship of PDW (a) and PAR (b) to cardiovascular events. The solid lines represent adjusted hazard ratio estimates, and the dashed lines represent 95% confidence intervals, respectively. Model adjusted for age, sex, smoking history, history of cardiovascular disease, diabetes mellitus, body mass index, systolic blood pressure, diastolic blood pressure, eGFR, hemoglobin, platelet, LDL-cholesterol, proteinuria, use of ACE inhibitor or ARB, and use of antiplatelet agent. PDW, platelet distribution width; PAR, PDW-to-albumin ratio; eGFR, estimated glomerular filtration rate; LDL, low-density lipoprotein; ACE, angiotensin-converting enzyme; ARB, angiotensin II receptor blocker.

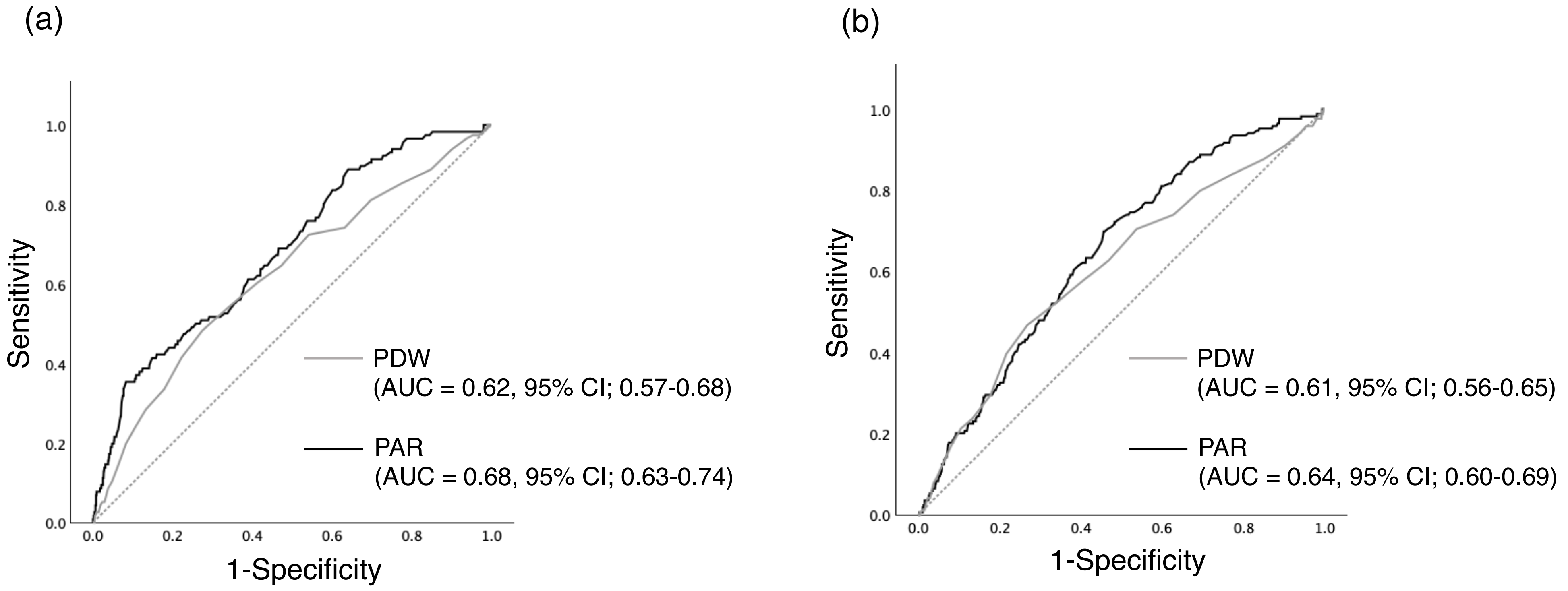

Figure S5. Comparison of the ROC curves of PDW and PAR for all-cause death (a) and cardiovascular events (b) in patients with hypertension. PDW, platelet distribution width; PAR, PDW-to-albumin ratio. AUC, area under the curve; CI, confidence interval; ROC, receiver-operating characteristic.
